# Adaptive Expertise in Continuing Pharmacy Professional Development

**DOI:** 10.3390/pharmacy8010021

**Published:** 2020-02-18

**Authors:** Naomi Steenhof

**Affiliations:** 1 Leslie Dan Faculty of Pharmacy, University of Toronto; Toronto, ON M5S 3M2, Canada; naomi.steenhof@utoronto.ca; Tel.: +1-416-978-2341; 2 The Wilson Centre, University Health Network and University of Toronto; Toronto, ON M5G 2C4, Canada

**Keywords:** continuing professional development, pharmacy education, adaptive expertise

## Abstract

Pharmacists are facing rapid changes and increasing complexity in the workplace. The astounding rate of both the evolution and the development of knowledge in pharmacy practice requires that we develop continuing professional development (CPD) to foster and support innovation, creativity, and flexibility, alongside procedural expertise. Adaptive expertise provides a conceptual framework for developing experts who can both perform professional tasks efficiently as well as creatively handle new and difficult-to-anticipate problems. This article approaches knowledge production in daily pharmacy practice and CPD through a cognitive psychology lens, and highlights three educational approaches to support the development of adaptive expertise in the workplace: (1) explaining not just what to do, but why you are doing it, (2) allowing and encouraging struggle, and (3) asking “what if” questions to encourage meaningful variation and reveal underlying core concepts. These three evidence-based strategies will cultivate long-term learning and will support pharmacists as we move into more complicated and ambiguous roles. Pharmacy CPD can be transformed to support the development of both procedural and conceptual knowledge in a local environment to support learning and innovation.

## 1. Introduction

Smita owns a pharmacy in a small town in Ontario. She has just hired Jon, a new pharmacy graduate, to work in her store. Smita is anticipating that if Jon is a good fit that she will slowly start reducing her hours as a form of semiretirement.

On Jon’s sixth week, a patient’s daughter drops off a prescription for her 75-year-old mother who has just undergone elective hip surgery and is being discharged from the hospital that day. Jon speaks to her daughter for a little while, before letting her know that the prescription will be ready later that morning. The prescription is for hydromorphone 2 mg po q4h prn, a total of 200 tablets. “Wow, that’s a lot of hydromorphone”, Jon says. “Do you think this looks ok for us to dispense?” 

Smita feels irritated. Jon is constantly asking for her advice and approval to dispense medications, and after six weeks she is expecting more independence from him. At this point, Smita is unsure what to do. She tells Jon that she thinks that both the dose and the quality are high for the patient. Smita tells Jon that she will call the patient to find out how much pain medication she was using in the hospital and see whether she is comfortable with trying acetaminophen and a lower dose of the hydromorphone. Jon nods, says thank you, and then goes out to the local coffee shop to pick up a morning coffee for the two of them.

Smita feels so disappointed. She thought Jon was going to be a great hire, and he showed so much promise during the interview. He just seems to be overwhelmed and, frankly, floundering. There was another candidate who seemed interested in the position, but Smita did not feel like that person was going to be as good of a fit.

*Is there something that Smita can do to help Jon onboard more successfully before she starts to look for another pharmacist to hire?*

There are many possible reasons why Jon did not want to make a decision and why he seemed reluctant to engage with the problem. Even though Jon just graduated from pharmacy school, he probably does not remember learning about this exact situation in his formal classes. If you spoke to Jon’s professors, they would say they taught the material; however, Jon likely does not have a strong sense of how the concepts he was taught are connected (i.e., he has weak conceptual connections). Additionally, while he had a clinical placement in an internal medicine unit, he is having difficulty connecting the principles and concepts from this placement to this new community pharmacy practice context. Jon may also have a half-formed idea of what he should do for this patient, but it is easier for him to ask Smita for permission.

Jon has been taught a lot of information in pharmacy school, but he still needs to accumulate practical experience to consolidate and restructure his knowledge. This experience will now mostly consist of solving problems in Smita’s community pharmacy. So, what strategies can Smita use to help Jon develop his expertise as he continues to work in her pharmacy?

## 2. Rapid Changes

The health care environment is experiencing rapid transformation all over the world in response to environmental pressures, shifting pathologies, and shifting political priorities. In addition to this rapid change, there is also increasing complexity in the work environment due to higher levels of required knowledge and task variety [1,2]. These rapid changes in the health-care system have compelled pharmacists to change their professional role [3]. Pharmacy practitioners are expected to provide care through expanded patient-focused clinical activities in a variety of health care settings, including providing comprehensive medication reviews, providing prescribing recommendations, providing preventative care services, and providing targeted clinical services. While pharmacists have always had to adapt to uncertain and complex situations, it is now even more important that pharmacists be innovative and flexible to solve new problems in their everyday practice [4]. The challenge facing pharmacists (and all other health care professionals) is to develop educational strategies that foster innovation, creativity, and flexibility, alongside procedural competency in an increasingly complex workplace environment.

## 3. Adaptive Expertise 

Smita wants to hire a pharmacist who can not only efficiently manage the routine, everyday problems that come up in her community pharmacy practice but is also able to generate solutions to new problems. This type of expertise is known as adaptive expertise, and goes beyond traditional models of expertise to emphasize the ability to creatively handle new and difficult-to-anticipate problems [5]. Hatano and Inagaki developed the adaptive expertise framework to explore and explain differences in experts’ ability to transfer learning, which is an essential element of efficiency and innovation. 

Hatano and Inagaki defined routine experts as those who developed a high but narrow procedural proficiency, for example, a worker in a fast-food restaurant. Routine experts are taught to focus on and become very efficient at following a set routine without necessarily being able to explain why they are doing specific actions. Routine expertise can be good; however, it can also lead to cognitive entrenchment, where people over apply or misapply old strategies to new situations [6]. 

In contrast, adaptive experts are not only able to perform tasks efficiently but also can flexibly and creatively solve new problems [7]. Adaptive experts can understand why specific ways of doing things work, can vary their approach when needed, and can invent new procedures when none of the known procedures are effective. Knowing when and how to transfer knowledge to a new situation helps to avoid cognitive fixedness [8].

Hatano and Inagaki described three qualities of adaptive experts: the ability to articulate the principles that underlie their skills, the ability to judge conventional and nonconventional versions of skills, and the ability to alter skills according to contextual changes [5]. Bransford and Schwartz added a fourth quality to this list: the ability to learn from new situations and avoid over-applying schemas [7]. 

The question for pharmacists and pharmacy educators is, of course, how to develop adaptive expertise in learners. Hatano and Inagaki proposed three environmental factors that influence this development: a workplace that has built-in unpredictability and allows a person to have a significant variation in the practice of their skills, the degree to which people can approach a task in a variety of ways, and the degree to which the organizational culture emphasizes understanding a task versus prompt performance. Community pharmacies contain all three environmental factors, which could positively influence the development of adaptive expertise. However, the tendency for some community pharmacies to foster an efficiency-oriented environment may influence learners and pharmacists toward routine expertise.

## 4. Knowledge Production

Often, when we think of continuing professional development (CPD), we think of programs and education innovations that are produced by universities and external companies; however, this marginalizes the value and impact of the knowledge that is produced by pharmacists in the course of their daily practice [4]. This knowledge production takes place every day in community pharmacies when pharmacists and pharmacy technicians work to adapt their practice to the challenges of their environment, including issues of patient management, organizational efficiency, and collaboration with other health care providers [4]. Smita has pharmacy practice and management expertise, and has developed innovations that allow her practice to thrive and serve patients in her community. Smita needs to be able to pass this knowledge, these skills, and these abilities on to Jon. Unfortunately, the formal education and CPD that Jon has access to through pharmacy associations and professional regulatory bodies may not contain everything Jon needs to learn to thrive in this position. Using an adaptive expertise framework for onboarding Jon would allow Smita to implement some principles from cognitive psychology and health profession education that would help Jon thrive in his new workplace.

Here are three suggestions that Smita can implement to help scaffold Jon’s learning and prepare him to practice pharmacy safely and effectively. These strategies will support Jon’s continued growth into a practitioner who Smita will eventually be able to trust to take over her pharmacy practice in her community. All pharmacists, including Smita, can rely on these strategies without requiring formal CPD products.

## 5. Strategies

### 5.1. Asking and Explaining “Why” Questions

When Smita is resolving a problem in the pharmacy, she should explain to Jon not just what she is doing (supporting procedural knowledge) but why she is doing it (supporting conceptual knowledge). Often during on-the-job instruction, trainers will explain exactly what to do in a specific situation. This immediate instruction is valuable for developing routine expertise if the person comes across the same situation in the future, but will not help when the person is faced with a variant of that condition where they need to transfer knowledge into that new situation. In a work-place environment where pharmacists become very familiar with repeated skills, it is possible to be effective most of the time with just procedural skills. However, procedural efficiency is only useful for a limited set of problems, because it is difficult for the pharmacist who is a routine expert to recombine the information embedded in the skill in a new way [5].

Conceptual knowledge underlies expertise development and skills transfer and refers to the generalizable principles that transcend specific contexts of a task. Conceptual knowledge is sometimes described as “knowing why” [9]. The training designed at adaptive expertise development requires that learners must be forming conceptual knowledge (“knowing why”) while at the same time learning procedural efficiency (“knowing how”). Explaining why a procedure works will help Jon to build the conceptual knowledge that he can rely on the next time he comes across a novel problem. These “why” questions will prompt Jon to make a mechanistic connection—i.e., understand the reason why a medication is working in a specific way—and create meaningful connections to the underlying pharmacological mechanism to help Jon understand the underlying principles of specific concepts. This, in turn, will shift the emphasis from surface, routine understanding to deeper elements that will lead to the creation of conceptual knowledge. 

Example: Smita could explain to Jon why she wants to follow-up on the dose and frequency of the hydromorphone. She could explain that hydromorphone is a very potent opioid, and the difference between 1mg and 2 mg is significant, especially in most opioid-naïve 75-year-old women. One of the consequences of too high of a dose of hydromorphone is increased drowsiness, which could lead to increased risk of falls and/or decreased participation in rehabilitation post-surgery. The reason that opioids, like hydromorphone, increase the risk of falls is partly because of anticholinergic activity in the central nervous system. Giving the patient the option of 0.5–1 mg dosage will give them flexibility in managing their pain while being safer for the patient which will support their recovery.

### 5.2. Allow and Encourage Struggle

Smita should allow Jon to struggle for a little while with a problem, instead of immediately giving him the answer. Jon may not generate the correct solution immediately, but trying to come up with an answer may activate prior knowledge which can improve his conceptual understanding and support later learning. Jon may also be more ready and better prepared to learn from Smita’s instruction [10]. Struggling is an important tool that will support the development of adaptive expertise [11]. Once Jon has struggled to come up with a solution, he may make some mistakes. This is an important time for Smita to give feedback and share her established approach to solving a problem (both the “what” and the “why”). Smita can also reemphasize important principles, highlight important features, reinforce performance, and correct conceptual errors. Research in the learning sciences suggests that struggling (followed by instruction) can help learners better notice and attend to the significant features of the new concept [12]. The struggle and invention that occurs during problem-solving may be crucial if the goal is to develop a deeper conceptual understanding of pharmacy practice which will help him solve problems in the future [10].

Example: Smita could have allowed Jon to try to resolve the hydromorphone issue on his own, and when he realized that he needed more information she could have then stepped in to help fill in the gaps. Experiencing struggle will allow Jon to recognize his limitations and how to utilize outside resources when he is unsure how to proceed. Even though Jon may have realized that 200 tablets was too large of a quantity postsurgery and may have called the hospital to ask about a smaller dose, he may not have realized that the 2 mg dose was potentially too high. However, it would have been better if Jon had gotten to the point of dispensing the prescription before Smita stepped in to talk through a solution with him to ensure that the struggle was productive.

### 5.3. “What If” Questions

Another strategy Smita can use to help encourage variation for Jon is to ask him "what if" questions to emphasize the core clinical concept that he is struggling with [13]. Asking "what if" questions will allow Smita to vary aspects of a situation that they may not come across while they are working together. These questions will reinforce the key elements of the concept that he is learning and will push Jon to extend his understanding of the underlying mechanisms of the clinical concept. Asking “what if” questions will also help John discern critical differentiating features that he might otherwise overlook [14]. When Jon starts to understand the deeper, unchanging principles he will more likely be able to create a mental model and more readily transfer this knowledge into a new situation [15]. 

To do this, Smita must elucidate what the important clinical concept is that Jon is misunderstanding. For example, in this case, the core clinical issue goes beyond simply not dispensing too much hydromorphone. The deeper concepts are related to effective and safe opioid use for opioid-naïve patients. She can then strategically ask “what if” questions that vary the context and surface presentation.

Example: Once Jon is confident that he has made the right decision concerning the hydromorphone prescription, Smita could ask Jon "what if the patient was 45 years old?” or “What if the patient was being discharged after a craniotomy?” Would that change the questions you would ask the patient or how you would approach the situation?" This variation would emphasize the core clinical concept (safe use of opioids) as opposed to focusing on the “correct” dose of hydromorphone following elective hip surgery in a 75-year-old female patient.

## 6. Summary

These three strategies, rooted in the cognitive psychology literature, will cultivate long-term learning and help Smita train Jon throughout the next few years as she prepares to retire and sell her pharmacy practice to Jon. The importance of CPD that is rooted in evidence is imperative for the evolution of the procession as pharmacists move into more complicated and ambiguous roles. Pharmacy CPD can be transformed to support the development of both procedural and conceptual knowledge in a local environment to support learning and innovation.

**Strategies for CPD providers**

Rapid changes in the health care environment have compelled pharmacists to change their professional role. Pharmacy practitioners are providing expanded patient-focused clinical activities in a variety of settings, and they must be innovative, creative, and flexible to solve new problems that will arise in everyday practice.

Adaptive expertise is a framework that emphasizes both performing tasks efficiently and the ability to creatively handle new and difficult-to-anticipate problems

Three strategies to foster adaptive expertise for continuing professional development are as follows:

1. Explaining not just what to do, but why you are doing it;

2. Allowing and encouraging new practitioners to struggle, and following-up with direct instruction;

3. Asking “what if” questions to encourage meaningful variation and reveal the underlying core clinical concepts.
